# Needs and preferences of women with prior severe preeclampsia regarding app-based cardiovascular health promotion

**DOI:** 10.1186/s12905-022-02004-5

**Published:** 2022-10-29

**Authors:** Lili L. Kókai, Marte F. van der Bijl, Martin S. Hagger, Diarmaid T. Ó Ceallaigh, Kirsten I.M. Rohde, Hans van Kippersluis, Alex Burdorf, Johannes J. Duvekot, Jeanine E. Roeters van Lennep, Anne I. Wijtzes

**Affiliations:** 1 Department of Public Health, Erasmus MC University Medical Center, Rotterdam, the Netherlands; 2 Department of Psychological Sciences, University of California, Merced, CA USA; 3 Faculty of Sport and Health Sciences, University of Jyväskylä, Jyväskylä, Finland; 4 Erasmus School of Economics, Erasmus University Rotterdam, Rotterdam, the Netherlands; 5 Tinbergen Institute, Erasmus University Rotterdam, Rotterdam, the Netherlands; 6 Tinbergen Institute and Erasmus Research Institute of Management, Erasmus University Rotterdam, Rotterdam, the Netherlands; 7 Department of Obstetrics and Gynecology, Erasmus MC University Medical Center, Rotterdam, the Netherlands; 8 Department of Internal Medicine, Erasmus MC University Medical Center, Rotterdam, the Netherlands; 9 P.O. Box 2040, 3000 CA Rotterdam, the Netherlands

**Keywords:** Preeclampsia, Cardiovascular health promotion, Intervention design, Needs and preferences assessment

## Abstract

**Background:**

Women with prior severe preeclampsia are at an increased risk for cardiovascular diseases later in life compared to women who had a normotensive pregnancy. The objective of this study was to assess their needs and preferences regarding app-based cardiovascular health promotion.

**Methods:**

Patients (*n* = 35) of the Follow-Up PreEClampsia Outpatient Clinic (FUPEC), Erasmus MC, the Netherlands, participated in an anonymous online survey. The main outcomes under study were women’s needs for health behavior promotion, and their preferences with respect to intervention delivery. Descriptive statistics were used to evaluate needs, and thematic analysis was used to analyze preferences.

**Results:**

Women’s primary need for health behavior promotion pertained to their fat and sugar intake and physical activity; for some, to their mental health (practices), fruit and vegetable intake, salt intake, and water intake; and for a few, to their alcohol and tobacco use. Most women preferred an app-based intervention to include, in descending order: the tracking of health-related metrics, an interactive platform, the use of behavior change strategies, the provision of information, and personalization.

**Conclusion:**

Cardiovascular health promotion targeting women with prior severe preeclampsia should feel relevant to its audience. App-based interventions are likely to be well received if they target fat and sugar intake and physical activity. These interventions should preferably track health-related metrics, be interactive, contain behavior change strategies, provide information, and be personalized. Adopting these findings during intervention design could potentially increase uptake, behavior change, and behavior change maintenance in this population.

**Supplementary Information:**

The online version contains supplementary material available at 10.1186/s12905-022-02004-5.

## Introduction

The leading cause of death in women worldwide are cardiovascular diseases (CVDs), contributing to 35% of female deaths [1]. Some risk factors for CVDs are unique to women, such as hypertensive disorders of pregnancy. These disorders affect 5 to 10% of pregnancies globally, and their prevalence is increasing [2, 3]. Of all hypertensive disorders of pregnancy, preeclampsia, which complicates 2 to 5% of all pregnancies [4], increases CVD risk the most: it has been linked to a two- to eightfold increased risk throughout the lifespan [5–7]. Maternal factors associated with an increased risk of preeclampsia are antiphospholipid antibody syndrome, prior preeclampsia, chronic hypertension, pre-gestational diabetes, and obesity [8]. Given that the causes and the early diagnosis of preeclampsia are not yet fully understood [4, 9], attention to the cardiovascular follow-up and risk management of women with prior preeclampsia is warranted [6, 7]. CVD risk can be decreased substantially by participating in healthy lifestyle behaviors [10, 11]. Therefore, efficacious health promotion interventions are warranted in this high-risk group [12].

Health behavior change interventions have been widely applied in the general population with demonstrable efficacy [13–17]. When it comes to the medium of interventions, web-based interventions have several advantages over face-to-face interventions: they are comparatively low cost, have a wide reach, and provide flexibility in intervention location and time [18, 19]. Web-based interventions were previously found to appeal to women with prior preeclampsia due to the flexibility they provide, i.e., they fit more easily into the demanding and unpredictable schedules of (often young and working) mothers [20–22]. Access to web-based interventions may be further enhanced by delivering them via mobile phone optimized web browsers or dedicated mobile apps, instead of desktop optimized web browsers, called mHealth [23]. Health apps are proliferating rapidly —there are now more than 350,000 available for download [24]. A prerequisite of health app use is owning a smartphone: about half of the world’s population [25] and 84% of the Dutch population meets this criterion [26]. Over half of Dutch women already use health apps, primarily to monitor their health behaviors, with another quarter being open to using one in the future [27].

It is increasingly recognized that to design an intervention that resonates with its intended audience, one should assess the needs and preferences of the study population prior to the development of the intervention protocol. This may increase intervention uptake, and enhance behavior change and maintenance [28]. Both quantitative and qualitative methods have been previously used to yield insight on needs and preferences, and to provide recommendations for the design of mHealth interventions in the general population [29], and in specific patient groups, such as hypertensive and CVD patients [30–32], and obstetric and gynecological patients [33–36]. In women with prior preeclampsia, previous research has offered insights into preferences for postpartum lifestyle counseling [21], the factors that influence the use of mHealth to monitor preeclampsia-related symptoms [37], and the acceptability of a specific web-based health promotion intervention [38]. To our knowledge, the needs of women with prior severe preeclampsia regarding the behavioral target of app-based cardiovascular health promotion, and their preferences for the delivery of such an intervention, have not been previously assessed.

With this study we aim to further understanding of the needs and preferences of women with prior severe preeclampsia for app-based cardiovascular health promotion. We define needs as the extent to which women struggle to participate in certain health-promoting behaviors, plan to make positive changes to these behaviors, and are interested in participating in an app-delivered program targeting these behaviors. More specifically, our objective is to gain insight into women’s needs regarding behaviors related to cardiovascular health, namely: physical activity, fat and sugar intake, fruit and vegetable intake, salt intake, water intake, mental well-being (practices), alcohol use, and tobacco use [11]. Our second aim is to understand women’s preferences regarding the delivery of app-based cardiovascular health promotion. Our related objective is to explore their wishes regarding app content, functionalities, and interface.

## Method

### Study setting

Study participants were recruited from an outpatient clinic for women with prior severe preeclampsia. In the Erasmus Medical Center (Erasmus MC), cardiovascular follow-up and risk management is provided for women with prior severe preeclampsia at the multidisciplinary Follow-Up PreEClampsia Outpatient Clinic (FUPEC), unique in the Netherlands [39]. Presently there are around 1500 patients enrolled at the clinic, with an additional 100 to 150 women registering each year.

### Study population

Participant recruitment at the FUPEC and online data collection took place between September and November 2020 (*n* = 35). Inclusion criterion for participation was having experienced severe preeclampsia at least once, as per the definition of the American Congress of Obstetricians and Gynecologists [40]. Exclusion criteria for participation were: <18 years of age, pregnant at time of inclusion, < 3 months after delivery, any circumstance preventing moderate-to-vigorous intensity physical activity (e.g., illness, injury, surgery, rehabilitation), no working knowledge of Dutch, and no possession of a smartphone. No upper age limit was applied. These exclusion criteria were employed to obtain a sample of women similar to those who will enroll in an app-based cardiovascular health promotion program [41]. A total of six women were excluded: three women were < 3 months after delivery, and three women had insufficient knowledge of Dutch. Invited women were informed that participation in the study was voluntary, and that they could withdraw at any point, without having to provide a reason. Women who chose to participate signed an informed consent form prior to participation. As is common practice for studies using qualitative methods, a specific target sample size was not pre-defined. Instead, recruitment was planned to be considered complete after data saturation was reached on the qualitative outcomes under study [42]. Responses received from participants were read while the online survey was still open for further recruitment. When, during this iterative process of data familiarization, study authors LLK and MFVDB agreed that new themes were not expected to arise from the inclusion of additional participants, the online survey was closed.

### Patient and public involvement

Patients and members of the public were not involved in the design, conduct, or reporting of this study.

### Design

An anonymous online survey was administered.

### Sampling strategy

The study used criterion sampling, i.e., participants had to have prior experience with severe preeclampsia [43].

### Procedure

Women were asked at their FUPEC consultation whether they were interested in joining the study. Women who did not show up at their scheduled consultation were asked via email. Those that expressed interest either at the consultation or via email received the survey. Of the 122 women asked, 119 agreed to receive the survey. Of these women, 55 started the survey, and 35 provided complete responses. Only complete responses were used in the current analyses. Women who did not provide complete responses (*n* = 20) were comparable to the study sample (*n* = 35) in for example age, educational level, and when they had experienced severe preeclampsia (data not shown). The survey assessed four topics: demographics, needs for app-based cardiovascular health promotion, perceived determinants of physical activity, and preferences for app-based cardiovascular health promotion. The current study used data on the first, second, and fourth topics. Data on the third topic was collected for the purpose of a qualitative assessment of physical activity determinants, the results of which will be published separately. The survey was hosted online on the data capture tool Limesurvey [44]. Data were imported into IBM SPSS Statistics and NVivo for analyses [45, 46].

### Main outcome measures

The main outcomes of this study were participants’ needs and preferences with respect to app-based cardiovascular health promotion. The recruitment materials and survey were developed by members of the research team, including JERVL and JJD as clinicians and MFVDB as medical student of the Follow-Up PreEClampsia Outpatient Clinic (FUPEC). Questions assessing needs were based on previous studies gauging the needs of a population prior to developing an mHealth intervention [30, 34], and were surveying health behaviors that are relevant for cardiovascular health promotion [11]. Questions assessing preferences were based on prior studies that examined the preferences of a population regarding content, functionality and interface before developing an mHealth intervention [30, 34, 35], and by the persuasive design framework of web-based interventions [47]. The questions have not been previously validated.

Participants answered one question each about the three components of needs: struggling to participate in certain health-promoting behaviors, planning to make positive changes to these behaviors, and being interested in participating in an app-delivered program targeting these behaviors. Spearman’s rho correlation analyses were performed between the three items for each health behavior to support their validity as positively related, but distinct components (for coefficients and significance levels see supporting information, supporting Table 1 ). We assessed needs regarding physical activity, fat and sugar intake, fruit and vegetable intake, salt intake, water intake, mental well-being (practices), alcohol use, and tobacco use [11]. First, participants reported on their struggle to follow a healthy lifestyle concerning these behaviors (e.g., “How often do you struggle to make healthy choices when it comes to fat and sugar intake?”) on a seven-point scale (1 = very rarely to 7 = very often). Second, participants reported their behavior change intentions regarding these behaviors (e.g., “How often do you think of making positive changes to your physical activity?”) on a seven-point scale (1 = very rarely to 7 = very often). Last, participants reported their interest in partaking in an app-based intervention targeting these behaviors (e.g., “How interested would you be in partaking in an app-based intervention targeting fruit and vegetable intake?”) on a seven-point scale (1 = not interested to 7 = very interested). For questions about alcohol and tobacco use, the response option not applicable (N/A) was included to accommodate for women who do not engage in these behaviors. Participants were assumed to engage in all the other studied behaviors to some extent, therefore, the response option N/A was not added.

Participants also reported their preferences for the delivery of an app-based intervention. To this aim, participants responded to a series of open-ended questions assessing three aspects of intervention delivery: content (e.g., “What should this app contain?”), functionality (e.g., “What should this app do?”), and interface (e.g., “How and with whom would you like to communicate via the app?”). Participants also reported on the acceptable number of weeks and hours per week of the intervention (“Time demand: What do you think is reasonable?”).

Participants reported their demographic characteristics: age (years), number of children (number), living situation (with or without partner, with or without children), educational level (lower, middle, higher; classified using the International Standard Classification of Education [48]), paid employment status (yes, no; if yes, number of hours per week), when they had experienced severe preeclampsia (between three months and one year ago; between one and three years ago; over three years ago), and whether preeclampsia-related health complaints were still present (yes, no; if yes, what complaints).

### Data analysis

Participants’ demographic characteristics, and responses on scaled items used to identify the needs of the population in terms of health promotion target behavior were reported using descriptive statistics. Scale ratings between 1 and 4 were collapsed into *No*, and ratings between 5 and 7 were collapsed into *Yes*. For alcohol and tobacco use, N/A was collapsed into *No* as well.

Thematic analysis was used to identify themes in participants’ preferences regarding intervention delivery [49–51]. Inductive content analysis for emergent themes was applied, consistent with guidelines for the analysis of qualitative data using the grounded theory approach [52]. After reading and re-reading participants’ responses, LLK and MvdB defined coding instances, and identified five recurring themes in these instances. They then returned to the data independently and categorized each coding instance into one of the five themes. A small number of coding instances were categorized as belonging to two themes (for examples of the thematic analysis procedure see supporting information, supporting Table 2). Initial interrater percent agreement was 91%. Subsequently, categorizations were revisited until 100% agreement was reached.

## Results

### Characteristics of the study population

Table 1 shows the characteristics of the study population (*n* = 35). Participants had a median age of 35 years. Most women had one child (54%) and were living with a partner (80%). The majority were highly educated (80%) and in paid employment (80%), working a median of 28 h per week. Most women experienced severe preeclampsia more than three years ago (54%). Half of women were still experiencing health complaints related to preeclampsia (49%), such as fatigue and anxiety, and problems with concentration and memory (examples of participants’ complaints are published under supporting information, supporting Table 3).

**Table 1:** Characteristics of the study population

Demographic characteristics		(*n* = 35)
Age*	Years	35 [32, 44]
Number of children	0 1 2 3	2 (6%) 19 (54%) 12 (34%) 2 (6%)
Living situation	With partner and children Without partner, with children With partner, without children Without partner and children	26 (74%) 7 (20%) 2 (6%) 0 (0%)
Educational level**	Lower Middle Higher	0 (0%) 7 (20%) 28 (80%)
Paid employment	Yes No If yes, hours/week*	28 (80%) 7 (20%) 28 [20, 32]
**Preeclampsia characteristics**		
Time since severe preeclampsia	≥ 3 months to 1 year 1–3 years ≥ 3 years	8 (23%) 8 (23%) 19 (54%)
Preeclampsia-related health complaints still present	Yes No	17 (49%) 18 (51%)

Displayed value is frequency (percentage of total participants) unless marked with a *, in which case the displayed value is the median [20, 32, 44]]

**Classified using the International Standard Classification of Education

### Needs regarding health promotion target behavior

Table 2 shows the needs of participants in terms of the target behavior of the intervention. In descending order, participants struggled to follow a healthy lifestyle with respect to their fat and sugar intake (43%), physical activity (31%), water intake (20%), mental well-being (practices) (17%), salt intake (15%), alcohol use (11%), fruit and vegetable intake (6%), and tobacco use (6%). Results regarding planning to make positive changes to these behaviors, and being interested in participating in an app-based intervention targeting these behaviors showed a similar pattern; although generally, more women reported planning to make positive changes and being interested in an app-based intervention, than they reported struggling with health behaviors.

**Table 2:** Needs regarding health promotion target behavior

(*n* = 35)		Struggling to follow a healthy lifestyle regarding…	Planning to make positive changes to…	Interested in participating in intervention targeting…
*Physical activity*	*Yes* *No*	11 (31%) 24 (69%)	16 (46%) 19 (54%)	17 (49%) 18 (51%)
*Fat and sugar intake*	*Yes* *No*	15 (43%) 20 (57%)	22 (63%) 13 (37%)	17 (49%) 18 (51%)
*Fruit and vegetable intake*	*Yes* *No*	2 (6%) 33 (94%)	13 (37%) 22 (63%)	10 (28%) 25 (72%)
*Salt intake*	*Yes* *No*	5 (15%) 30 (85%)	9 (25%) 26 (75%)	9 (25%) 26 (75%)
*Water intake*	*Yes* *No*	7 (20%) 28 (80%)	11 (31%) 24 (69%)	7 (20%) 28 (80%)
*Mental well-being (practices)*	*Yes* *No*	6 (17%) 29 (83%)	15 (43%) 20 (57%)	12 (34%) 23 (66%)
*Alcohol use*	*Yes* No*	4 (11%) 31 (89%)	4 (11%) 23 (89%)	3 (9%) 25 (91%)
*Tobacco use*	*Yes* No*	2 (6%) 7 (94%)	2 (6%) 5 (94%)	1 (3%) 11 (97%)

Displayed value is frequency (percentage of total participants).Scale ratings between 1 and 4 were collapsed into *No*, and ratings between 4 and 7 were collapsed into *Yes*.* The option not applicable (N/A) was included for alcohol and tobacco use. N/A was collapsed into *No* as well: 15% and 74% reported N/A for struggling with the behavior, 23% and 80% reported N/A for planning to make positive changes to the behavior, and 20% and 66% reported N/A for being interested in an intervention concerning alcohol and tobacco use, respectively

### Preferences regarding intervention delivery

Table 3 shows the five themes of preferred intervention delivery, in descending order: tracking of health-related metrics (i.e., monitoring outcomes over time), interactivity (i.e., two-way communication with other people or app), behavior change strategy (i.e., methods to alter determinants of behavior), information (i.e., health-related information), and personalization (i.e., tailored delivery). Example quotes of each theme are presented below in English (example quotes are published in their original language under supporting information, supporting Table 4). Table 3 also summarizes participants’ preferred intervention duration: 12 weeks (interquartile range 5 to 52 weeks), 2 h and 45 min per intervention week (interquartile range 1 to 5 h).

**Table 3:** Preferences regarding intervention delivery

Themes*	(*n* = 35)
Tracking	31 (89%)
Interactivity	26 (74%)
Behavior change strategy	24 (69%)
Information	20 (57%)
Personalization	19 (54%)
Preferred time demand**	
Time, number of weeks (*n* = 23)	12 [5, 52]
Time, hours per week (*n* = 32)	2.75 [1, 5]

*Displayed value is frequency (percentage of total participants)

**Displayed value is median [1, 5, 52]. Not all women reported a meaningful number to this question, therefore *n* < 35

### Tracking

The majority of participants (89%) mentioned the tracking of various health-related metrics as a preferred component of the intervention, for example “Measurement of steps, heart rate, exercise intensity”, “Tracking nutrition”, and “Monitoring well-being”.

### Interactivity

Three out of four women (74%) preferred the program to contain interactive elements, such as “Exercising together remotely”, “Points for exercise and drinking [water], competition with participants”, and “Asking questions to specialists and be able to approach fellow [preeclampsia] sufferers”.

### Behavior change strategy

Nearly three-quarter of participants (69%) mentioned behavior change strategies that they would like the app to include, for example “Tips on how to build up a daily routine”, “Amount of exercise per day/week and intervene accordingly: stimulate if it is not enough, reward if it is sufficient” (*Note: this coding instance was also coded as another theme, Tracking*), and “Tips (exercises, e.g., meditation) for reducing stress, busy mind, relaxation”.

### Information

Over half of women (57%) reported provision of information to be a desired element of the intervention, such as “Lots of information, but not just to ‘scare’ you, as in, *if you don’t move, you get this disease*! Instead, for example, *it has been proven that if you exercise X times a week, your blood pressure drops by X*. Digestible, smaller bits of information”, “Relationship between preeclampsia and exercise, and what effects this can have”, and “High blood pressure in combination with exercise, how much do you have to sweat or be out of breath, what is enough in terms of amount of exercise. Which exercises can help with certain complaints, which exercises help to create a basic level of fitness and how do you train from there. What food can you eat before, during and after exercise”.

### Personalization

Over half of participants (54%) preferred the program to be personalized, for example “Reminder of exercises, goals; compliments on results/knowledge/overview” (*Note: this coding instance was also coded as another theme, Interactivity*), “Enough choices to turn things on and off”, and “During the recovery process, I would have liked to have received feedback about which aspects were ‘normal’, and which need more attention or patience, and how to deal with them”.

## Discussion

The objective of this study was to identify the needs and preferences of women with prior severe preeclampsia for app-based cardiovascular health promotion. Women’s primary need for health behavior promotion pertained to their fat and sugar intake and physical activity; for some, their mental health (practices), fruit and vegetable intake, salt intake, and water intake; and for a few, their alcohol and tobacco use. Most women preferred the intervention to include, in descending order: the tracking of health-related metrics, an interactive platform, the use of behavior change strategies, the provision of information, and personalization.

### Interpretation of key findings

Our results indicate that women’s primary need lied in addressing their fat and sugar intake and physical activity. Both of these behaviors are closely linked to CVD risk, emphasizing the need for interventions that target these behaviors in this priority population [6, 11]. Participants’ interest in improving these behaviors could be due to their awareness of their heightened CVD risk [53, 54], further strengthened by their wish to provide a healthy environment to their children [55]. Previous research showed that women with prior preeclampsia wish to receive support in adopting a healthy lifestyle [21], and that their post-partum period is a window of opportunity for behavior change [56].

After their need to address fat and sugar intake and physical activity, women’s following priority was to gain better means to manage their mental health: half of participants reported to still experience health complaints related to preeclampsia, such as fatigue and anxiety, and problems with concentration and memory. Previous research has identified a negative impact of preeclampsia on mental health [57, 58]. A healthy lifestyle, such as engaging in physical activity, has been linked to improved mental health, therefore, future interventions should target multiple needs simultaneously [59–61].

Participants’ need to address their fruit and vegetable intake, salt intake and water intake was modest, and their need to address alcohol and tobacco use was low. Implementing interventions targeting these behaviors in this group may yield low uptake and little behavior change.

We found that women with prior severe preeclampsia have a desire to gain information as part of a cardiovascular health promotion program, further emphasizing that providing informational lifestyle counselling is consistent with patients’ preferences [22, 53]. Some women wanted to receive information on the relationship between preeclampsia, lifestyle behaviors, and CVD risk. Therefore, clinicians might want to consider devoting more time to elaborating on evidence-based recommendations to manage CVD risk after severe preeclampsia through the adoption of preventive health behaviors [6]. Moreover, participants were interested in receiving more detailed information on the interrelation between different health behaviors such as diet and physical activity, and how certain physical or mental health complaints may be alleviated. Therefore, informational intervention content could be enriched by consulting various specialists, such as dieticians, physiotherapists, or psychologists.

Additionally, participants preferred to receive more than ‘just information’: they were open to receiving instruction on behavior change strategies, such as planning, incentives, and stress reduction [62]. The primary preference of participants was the tracking of their health-related metrics, such as dietary intake, physical activity intensity, and mental well-being. This finding is in line with studies demonstrating that patient autonomy is an integral part of the successful self-management of chronic diseases [63]. Interactivity was also a prominent preference of our participants: for example, they wanted to use the app to communicate with specialists, and to chat with and exercise together with other women who had severe preeclampsia. Interactive game-like elements were also described, such as the collection of points and competition with other participants. Finally, our participants described a wish for the intervention to contain personalized elements, such as the option to customize content and the provision of feedback. These results, and previous findings that these intervention elements can enhance intervention effects and user usage and adherence, suggest that future app-based programs aimed to improve cardiovascular health in women with prior severe preeclampsia would benefit from including such elements in their delivery [64, 65].

### Strengths and limitations

The current study has several strengths. It is the first study to conduct an in-depth assessment of the needs and preferences of women with prior severe preeclampsia for app-based cardiovascular health promotion. Secondly, it yields several applicable suggestions for intervention researchers to inform the design of apps for women with prior severe preeclampsia, potentially increasing intervention uptake, behavior change, and behavior change maintenance. Finally, our findings could be applicable to other populations, such as women with a history of other types of hypertensive pregnancy disorders, or other pregnancy complications, such as intrauterine growth restriction or gestational diabetes. However, our study also had some limitations that should be taken into account when interpreting the results. It could be that as our study population was drawn from an outpatient clinic specialized in the cardiovascular follow-up and risk management of women with prior severe preeclampsia, participants had a higher awareness of their increased risk for CVDs than most women with prior preeclampsia. Second, some participant quotes offered little context, limiting the interpretation of preferences (e.g., participants did not specify whether a behavior change strategy would be useful in all health behavior contexts, or only the context they used to exemplify the strategy). Third, our study population was highly educated, limiting the generalizability of our findings to all socioeconomic groups, e.g. in terms of preferences regarding health apps. Fourth, the size of our study sample might have been too small to allow for generalizations to be made based on our quantitative findings, i.e. regarding needs. Fifth, while the experience of medical staff has provided some input on the comprehensibility and acceptability of our study materials, future studies should also including members of the target population in the pilot testing phase to garner external and lay perspectives. Finally, our study did not assess the extent to which self-perceived need for behavior change reflects actual unhealthy behavioral habits, i.e., quantitative data on participants’ health behavior was not collected, nor were participants informed of ideal values of all health behaviors under study.

## Conclusion

Cardiovascular health promotion targeting women with prior severe preeclampsia should feel relevant to its audience. App-based interventions are likely to be well received if they target fat and sugar intake and physical activity. These interventions should preferably track health-related metrics, be interactive, contain behavior change strategies, provide information, and be personalized. Adopting these findings during intervention design could potentially increase uptake, behavior change, and behavior change maintenance in this population.

## Electronic supplementary material

Below is the link to the electronic supplementary material.

Supplementary Material 1: Correlations between components of needs per health behavior

## Data Availability

The datasets generated and/or analyzed during the current study are not publicly available because participants were not asked to provide informed consent for the sharing of their data with third parties, but are available from the corresponding author on reasonable request.
