# Correction: Needs and preferences of women with prior severe preeclampsia regarding app-based cardiovascular health promotion

**DOI:** 10.1186/s12905-022-02133-x

**Published:** 2023-01-09

**Authors:** Lili L. Kókai, Marte F. van der Bijl, Martin S. Hagger, Diarmaid T. Ó. Ceallaigh, Kirsten I. M. Rohde, Hans van Kippersluis, Alex Burdorf, Johannes J. Duvekot, Jeanine E. Roeters van Lennep, Anne I. Wijtzes

**Affiliations:** 1 Department of Public Health, Erasmus MC University Medical Center, Rotterdam, The Netherlands; 2 Department of Psychological Sciences, University of California, Merced, CA USA; 3 Faculty of Sport and Health Sciences, University of Jyväskylä, Jyväskylä, Finland; 4 Erasmus School of Economics, Erasmus University Rotterdam, Rotterdam, The Netherlands; 5 Tinbergen Institute, Erasmus University Rotterdam, Rotterdam, the Netherlands; 6 Tinbergen Institute and Erasmus Research Institute of Management, Erasmus University Rotterdam, Rotterdam, The Netherlands; 7 Department of Obstetrics and Gynecology, Erasmus MC University Medical Center, Rotterdam, The Netherlands; 8 Department of Internal Medicine, Erasmus MC University Medical Center, Rotterdam, The Netherlands; 9 P.O. Box 2040, 3000 CA Rotterdam, The Netherlands

**Correction to: BMC Women's Health (2022) 22:427** 10.1186/s12905-022-02004-5

Following publication of the original article [1], the author has inserted the reference 66, and the same has been listed below:

66. World Medical Association. World Medical Association Declaration of Helsinki: ethical principles for medical research involving human subjects. JAMA. 2013;310(20):2191–2194. 10.1001/jama.2013.281053.

Also, the in text citation has been changed in the original article.

The original article has been corrected.
