# Supplementary material for: Needs and preferences of women with prior severe preeclampsia regarding app-based cardiovascular health promotion
Source: BMC Womens Health. 2022 Oct 29;22:427. doi: 10.1186/s12905-022-02004-5 (PMC9618195; doi:10.1186/s12905-022-02004-5)
Supplement: Supplementary file 1 — Supplementary Material 1: Correlations between components of needs per health behavior [file 12905_2022_2004_MOESM1_ESM.docx]

**Supporting information**

**Supporting table 1. Correlations between components of needs per health behavior**

| **Physical activity (*n* = 35)** | *Struggling with behavior* | *Interested in intervention* |
| --- | --- | --- |
| *Planning change* | 0.36* | 0.51** |
| *Interested in intervention* | 0.23 | - |

| **Fat and sugar intake**  **(*n* = 35)** | *Struggling with behavior* | *Interested in intervention* |
| --- | --- | --- |
| *Planning change* | 0.36* | 0.40** |
| *Interested in intervention* | 0.42** | - |

| **Fruit and vegetable intake (*n* = 35)** | *Struggling with behavior* | *Interested in intervention* |
| --- | --- | --- |
| *Planning change* | 0.37* | 0.55** |
| *Interested in intervention* | 0.46** | - |

| **Salt intake (*n* = 35)** | *Struggling with behavior* | *Interested in intervention* |
| --- | --- | --- |
| *Planning change* | 0.47** | 0.47** |
| *Interested in intervention* | 0.41** | - |

| **Water intake (*n* = 35)** | *Struggling with behavior* | *Interested in intervention* |
| --- | --- | --- |
| *Planning change* | 0.42** | 0.40** |
| *Interested in intervention* | 0.56** | - |

| **Mental well-being (practices) (*n* = 35)** | *Struggling with behavior* | *Interested in intervention* |
| --- | --- | --- |
| *Planning change* | 0.46** | 0.54** |
| *Interested in intervention* | 0.70** | - |

| **Alcohol use (*n* = 27)** | *Struggling with behavior* | *Interested in intervention* |
| --- | --- | --- |
| *Planning change* | 0.55** | 0.53** |
| *Interested in intervention* | 0.59** | - |

| **Tobacco use (*n* = 7)** | *Struggling with behavior* | *Interested in intervention* |
| --- | --- | --- |
| *Planning change* | 1.00** | 0.63 |
| *Interested in intervention* | 0.63 | - |

Spearman’s rho coefficients under 0.4 represent a weak correlation, between 0.4 and 0.79 a moderate correlation, and between 0.8 and 1 a strong correlation.

For alcohol and tobacco use N/A was treated as system missing, therefore *n* < 35.

* = Statistically significant at *p* < 0.05, one-tailed; ** = Significant at *p* < 0.01, one-tailed.

**Supporting table 2. Thematic analysis procedure examples**

| **Quote:**  “Measuring heart rate, how many minutes you have been active, being able to score the activity yourself” |
| --- |
| **Coding instance identification:**  Measuring heart rate + how many minutes you have been active + being able to score the activity yourself = 3 coding instances in total |
| **Categorization of coding instance into theme:**  Measuring heart rate = Coding instance categorized as tracking  How many minutes you have been active = Coding instance categorized as tracking  Being able to score the activity yourself = Coding instance categorized as personalization |
|  |
| **Quote:**  “Amount of exercise per day/week and intervene accordingly: stimulate if it is not enough, reward if it is sufficient” |
| **Coding instance identification:**  Amount of exercise per day/week and intervene accordingly: stimulate if it is not enough, reward if it is sufficient = 1 coding instance in total |
| **Categorization of coding instance into theme:**  Amount of exercise per day/week and intervene accordingly: stimulate if it is not enough, reward if it is sufficient = Coding instance categorized as tracking *and* behavior change strategy |
| **Quote:**  “Incorporate into daily routine, exercises that you can do anywhere” |
| **Coding instance identification:**  Incorporate into daily routine + exercises that you can do anywhere = 2 coding instances in total |
| **Categorization of coding instance into theme:**  Incorporate into daily routine = Coding instance categorized as behavior change strategy  Exercises that you can do anywhere = Coding instance categorized as information |

**Supporting table 3. Examples of participant quotes: health complaints still present due to prior preeclampsia**

| **Quote in Dutch**  **(Original)** | **Quote in English**  **(Translation)** |
| --- | --- |
| “Hoofdpijn, prikkelgevoelig, angstig, soms moe” | “Headache, sensitive to stimuli, anxious, sometimes tired” |
| “Geheugen-, concentratie-, leer-, oriëntatieproblemen, zeer prikkelgevoelig, zeer vermoeid” | “Problems with memory, concentration, learning and orientation, very sensitive to stimuli, very tired” |
| “Hypertensie” | “Hypertension” |
| “Korte termijn geheugen” | “Short-term memory” |
| “Ik ben nog snel moe en heb minder energie” | “I am still quickly fatigued and have less energy” |
| “Snel overprikkeld, niet op woorden kunnen komen, mindere concentratie” | “Easily overstimulated, unable to find words, less concentration” |
| “Mijn mentale draagkracht en conditie zijn nog lager” | “My mental capacity and condition are still lower [than before preeclampsia]” |
| “Ik heb nog steeds een hoge bloeddruk” | “I still have high blood pressure” |
| “Vergeetachtig, traumatische beelden zien, warrig” | “Forgetful, seeing traumatic images, confused” |

**Supporting table 4. Examples of participant quotes: intervention delivery preferences**

| **Theme** | **Quote in Dutch**  **(Original)** | **Quote in English**  **(Translation)** |
| --- | --- | --- |
| Tracking | “Meten van stappen, hartslag, intensiteit van sporten” | “Measurement of steps, heart rate, exercise intensity” |
| Tracking | “Het bijhouden van voeding” | “Tracking nutrition” |
| Tracking | “Welzijn monitoren” | “Monitoring wellbeing” |
| Interactivity | “Samen sporten op afstand” | “Exercising together remotely” |
| Interactivity | “Punten bij beweging en [water] drinken, competitie met deelnemers” | “Points for exercise and drinking [water], competition with participants” |
| Interactivity | “Vragen stellen aan specialisten en lotgenoten kunnen benaderen” | “Asking questions to specialists and be able to approach fellow [preeclampsia] sufferers” |
| Behavior change strategy | “Tips over hoe iemand de dagelijkse routine inbouwt” | “Tips on how to build up a daily routine” |
| Behavior change strategy  (and Tracking) | “Hoeveelheid beweging per dag/week en hierop ingrijpen: stimuleren als het te weinig is, belonen als het voldoende is” | “Amount of exercise per day/week and intervene accordingly: stimulate if it is not enough, reward if it is sufficient” |
| Behavior change strategy | “Tips (oefeningen, bijvoorbeeld meditatie) voor het verminderen van stress, drukte in het hoofd, ontspanning” | “Tips (exercises, e.g., meditation) for reducing stress, busy mind, relaxation” |
| Information | “Veel informatie, maar niet alleen ‘bang maken’ als in: *als je niet beweegt, krijg je deze ziekte*! Wel bijvoorbeeld, *bewezen is dat als je X keer per week sport, je bloeddruk met X daalt*. Dus maak het behapbaar, kleinere stukjes info” | “Lots of information, but not just to 'scare' you, as in, *if you don't move, you get this disease*! Instead, for example, *it has been proven that if you exercise X times a week, your blood pressure drops by X*. Digestible, smaller bits of information” |
| Information | “Relatie tussen preeclampsie en beweging en welke effecten dit kan hebben” | “Relationship between preeclampsia and exercise, and what effects this can have” |
| Information | “Hoge bloeddruk in combinatie met sporten, hoeveel moet je zweten of buiten adem zijn, wat is genoeg qua hoeveelheid sporten. Welke oefeningen kunnen helpen bij bepaalde klachten, welke oefeningen helpen bij het creëren van een basisconditie en hoe ga je van daaruit verder trainen. Welke voeding kun je voor, tijdens en na het sporten innemen” | “High blood pressure in combination with exercise, how much do you have to sweat or be out of breath, what is enough in terms of amount of exercise. Which exercises can help with certain complaints, which exercises help to create a basic level of fitness and how do you train from there. What food can you eat before, during and after exercise” |
| Personalization  (and Interactivity) | “Herinnering aan oefeningen, doelen; complimenten over resultaten/ kennis/ overzicht” | “Reminder of exercises, goals, compliments on results/knowledge/overview” |
| Personalization | “Genoeg keuzes om zaken aan en uit te zetten” | “Enough choices to turn things on and off” |
| Personalization | “In hersteltraject had ik wel feedback willen krijgen over welke aspecten 'normaal' waren en welke meer aandacht of geduld behoeven en hoe daarmee om te gaan” | “During the recovery process, I would have liked to have received feedback about which aspects were 'normal', and which need more attention or patience, and how to deal with them” |
